# Radical scavenging and antioxidant activities of methanolic extracts from *Hypericum* species growing in Bulgaria

**DOI:** 10.4103/0973-1296.62889

**Published:** 2010-05-05

**Authors:** Dimitrina Zheleva-Dimitrova, Paraskev Nedialkov, Gerassim Kitanov

**Affiliations:** *Department of Pharmacognosy, Faculty of Pharmacy, Medical University of Sofia, Dunav str. 2, 1000 Sofia, Bulgaria*

**Keywords:** Antioxidant activity, flavonoids, *Hypericum*, radical scavenging, tannins

## Abstract

Thirteen ***Hypericum*** species growing in Bulgaria were investigated for free radical-scavenging activity, antioxidant activity, total tannins and total flavonoids contents. Methanolic extracts from the ***Hypericum*** species were analyzed for radical scavenging and antioxidant activities using DPPH-, ABTS- free radicals, total antioxidant activity and inhibition of lipid peroxidation by ferric thiocyanate (FTC) method. Butylated hydroxytoluene and ascorbic acid were used as positive controls. Methanolic extracts from ***H. cerastoides, H. perforatum*** and ***H. maculatum*** demonstrate the highest antioxidant activities and are potential sources of natural antioxidant compounds. The quantification of tannins and flavanoids were determined in ***Hypericum*** species using Folin-Chiocalteu reagent and AlCl3, respectively. The amounts of the tannins ranged from 1.30 ± 0.01 mg/100 g dw in H. elegans to 8.67 ± 0.02 g/100 g dw in ***H. perforatum***. The highest concentration of flavonoids was found in ***H. cerastoides*** (1.22 ± 0.02 g/100g dw), and the lowest amount was established in ***H. olympicum*** (0.20 ± 0.03 g/100g dw).

## INTRODUCTION

Free radical production and lipid peroxidation are involved in the pathogenesis of some chronic diseases, including atherosclerosis, cardiac and cerebral ischemia, neurodegenerative disorders, carcinogenesis, diabetes, and rheumatic disorders.[1–4] In recent years, there has been an increased interest in phenolic compounds derived for their possible health benefits. The anticarcinogenic, antimutagenic, and cardioprotective effects of phenolic compounds are reported to be generally associated with their antioxidant properties of eliminating free radicals and alleviating lipid peroxidation.[5]

The genus *Hypericum* L. is a large group of herbs or shrubs consisting of approximately 450 species in 36 sections.[6] *Hypericum* species have been known for their antidepressant, analgesic, spasmolytic, antiviral and wound healing effects for many years.[7,8] Phytochemical investigations have shown the presence of naphthodianthrones hypericin and pseudohypericin,[9] tannins, flavonoids, xanthones, benzophenones.[10–12] Numerous substances have been suggested to act as antioxidants. Various phenolic antioxidants such as flavonoids, tannins, coumarins and more recently procyanidins have been shown to scavenge radicals in a dose dependent manner and therefore are viewed as promising therapeutic drugs for free radical pathologies.[13] Flavonoids and tannins are the major plant compounds with antioxidant activity.[14] The aim of the current study is to evaluate the antioxidant property of methanolic extracts from *Hypericum* species, including scavenging activities against DPPH and ABTS radicals, total antioxidant activity and inhibition of lipid peroxidation. Moreover, the total flavonoids and tannins were quantified.

## MATERIALS AND METHODS

### Chemicals and reagents

1,1-Diphenyl-2-picrylhydrazyl (DPPH), Folin-Chiocalteu reagent and linoleic acid were purchased from Sigma, USA. Ferrous chloride, 2,2'-azinobis-(3- ethylbenzothiazoline-6-sulfonic acid) (ABTS), 2,4,6-tri(2-pyridyl)-s-triazine (TPTZ), butylated hydroxytoluene (BHT) and ascorbic acid were purchased from Fluka, Germany. Potassium persulfate and Trolox (6-hydroxy-2, 5, 7, 8-tetramethylchroman-2-carboxylic acid) were from Sigma-Aldrich USA. All the other chemicals used including the solvents were of analytical grade.

### Plant material and extraction

Aerial parts of *Hypericum* species used for this study were collected during the flowering season from wild habitats. Voucher specimens from all plants samples were deposited in the Herbarium of the Institute of Botany-Bulgarian Academy of Sciences (SOM)[Table 1].

**Table 1:** Collection locality, collection time and voucher specimen of the studied *Hypericum* spp.growing in Bulgaria

Taxon	Collection locality	Collection time	Voucher specimen
*Hypericum aucheri* Jaub. et Spach	Momchilgrad, Rhodope Mountains	24 June 2005	SOM 144291
*Hypericum barbatum* Jacq.	Kopitoto, Vitosha Mountains	12 June 2005	SOM 144292
*Hypericum cerastoides*(Spauch) N. Robson	Snezhanka, Pamporovo, Rhodope Mountains	30 June 2004	SOM-Co-1198
*Hypericum elegans* Stephan ex Willd	v. Balgarevo, Kavarna	06 July 2005	SOM 153305
*Hypericum linarioides* Bosse	Petrohan, Stara Planina	20 July 2005	SOM-Co-1196
*Hypericum maculatum* Crantz	Goli vrah, Vitosha Mountains	23 July 2007	SOM-Co-1195
*Hypericum montbretii* Spach	v. Trigrad, Rhodope Mountains	25 June 2004	SOM-Co-1200
*Hypericum olympicum* L.	Krumovgrad, Rhodope Mountains	24 June 2005	SOM 144295
*Hypericum perforatum* L.	v. Yagodina, Rhodope Mountains	15 June 2007	SOM 144303
*Hypericum richeri* Vill.	Vitosha Mountains	25 July 2004	SOM-Co-1202
*Hypericum rumeliacum* Boiss.	Golo Bardo, Pernik	11 June 2005	SOM-Co-1199
*Hypericum tetrapterum* Fries	Golo Bardo, Pernik	4 August 2004	SOM 144307
*Hypericum umbellatum* A. Kerner	Vitosha Mountains	11 July 2004	SOM 144309

Air-dried aerial parts were sequentially extracted with CH_2_Cl_2_ and then with MeOH. The MeOH extracts (0.01 mg dw/ml) were assayed to determine antioxidant activities.

### Quantification of tannins

The quantification of tannins in the aerial parts was performed according to the European Pharmacopoeia[15] involving Folin-Chiocalteu reagent and pyrogallol as standard. The analyses were carried out at 760 nm. All determinations were performed in triplicate (*n* = 3).

### Quantification of flavonoids

The content of the flavonoids in the aerial parts was established spectrophotometrically at 430 nm by creating a complex with AlCl_3_ according to the European Pharmacopoeia.[16] The content of flavonoids was calculated as hyperoside. The measurements were carried out using a Shimadzu UV-1203 spectrophotometer (Japan). All determinations were performed in triplicate (*n* = 3).

### Determination of antioxidant activity

#### DPPH radical-scavenging activity

Scavenging activity of *Hypericum* extracts against DPPH radical was assessed according to the method of Blois[17] with some modifications. Briefly, 1 ml of *Hypericum* extracts (0.01 mg dw/ml) was mixed with 4 ml of 0.005 mg/ml DPPH methanol solution. The reaction mixture was vortexed thoroughly and left in the dark at room temperature for 30 min. The absorbance of the mixture was measured at 517 nm. Ascorbic acid and BHT were used as references. The ability to scavenge DPPH radical was calculated by the following equation: DPPH radical scavenging activity $\left(\%\right)=\frac{{\text{Abs}}_{control}-{\text{Abs}}_{sample}}{{\text{Abs}}_{control}}$, where Abs_control_ is the absorbance of DPPH radical in methanol; Abs_sample_ is the absorbance of DPPH radical solution mixed with sample extract /standard. All determinations were performed in triplicate (*n* = 3).

### ABTS radical scavenging assay

For ABTS assay, the procedure followed the method of Arnao *et al*.[18] with some modifications. The stock solutions included 7 mM ABTS solution and 2.4 mM potassium persulfate solution. The working solution was then prepared by mixing the two stock solutions in equal quantities and allowing them to react for 14 h at room temperature in the dark. The solution was then diluted by mixing 1 ml ABTS solution with 60 ml methanol to obtain an absorbance of 0.706 ± 0.01 units at 734 nm using a spectrophotometer. Fresh ABTS solution was prepared for each assay. Plant extracts (1 ml) were allowed to react with 1 ml of the ABTS solution and the absorbance was taken at 734 nm after 7 min using a spectrophotometer. The ABTS scavenging capacity of the extract was compared with that of BHT and ascorbic acid and percentage inhibition calculated as ABTS radical scavenging activity $\left(\%\right)=\frac{{\text{Abs}}_{control}-{\text{Abs}}_{sample}}{{\text{Abs}}_{control}}$ where Abs_control_ is the absorbance of ABTS radical in methanol; Abs_sample_ is the absorbance of ABTS radical solution mixed with sample extract/standard. All determinations were performed in triplicate (*n* = 3).

#### Total antioxidant activity (ferric reducing antioxidant power, FRAP)

The FRAP assay was done according to the method of Benzie and Strain[19] with some modifications. The stock solutions included 300 mM acetate buffer (3.1 g C_2_H_3_NaO_2_ × 3H_2_O and 16ml C_2_H_4_O_2_), pH 3.6, 10 mM TPTZ (2, 4, 6-tripyridyl-s-triazine) solution in 40 mM HCl, and 20 mM FeCl_3_ × 6H_2_O solution. The fresh working solution was prepared by mixing 25 ml acetate buffer, 2.5 ml TPTZ solution, and 2.5 ml FeCl_3_ × 6H_2_O solution and then warmed at 37°C before using. *Hypericum* extracts (0.15 ml) were allowed to react with 2.80 ml of the FRAP solution for 30 min in the dark condition. Readings of the colored product (ferrous tripyridyltriazine complex) were then taken at 593 nm. The standard curve was linear between 0.015 and 0.15 mM Trolox. Results are expressed in mM TE/g dry mass. Additional dilution was needed if the FRAP value measured was over the linear range of the standard curve. All determinations were performed in triplicate (*n* = 3).

#### Determination of antioxidant activity in linoleic acid system by the FTC method

The antioxidant activity of *Hypericum* extracts against lipid peroxidation was measured through ammonium thiocyanate assay, as described by Takao *et al*.,[20] with some modifications. The reaction solution, containing 0.2 ml of 0.01 mg/ml *Hypericum* extract, 0.2 ml of linoleic acid emulsion (25 mg/ml in 99% ethanol) and 0.4 ml of 50 mM phosphate buffer (pH 7.4), was incubated in the dark at 40 °C. A 0.1 ml aliquot of the reaction solution was then added to 3 ml of 70% (v/v) ethanol and 0.1 ml of 30% (w/v) ammonium thiocyanate. Precisely 3 min after the addition of 0.1 ml of 20 mM ferrous chloride in 3.5% (v/v) hydrochloric acid to the reaction mixture, the absorbance of the resulting red color was measured at 500 nm. Aliquots were assayed every 24 h until the day after the absorbance of the control solution (without *Hypericum* extract) reached maximum value. Butylated hydroxytoluene (BHT) was used as positive control. All determinations were performed in triplicate (*n* = 3).

## RESULTS AND DISCUSSIONS

### Tannins and flavonoids content

The amount of tannins, measured by Folin-Ciocalteu method, was expressed as pyrogallol equivalent (PE) and ranged from 1.30 to 8.67 g/100 g dry weight (dw) [Table 2]. The total flavonoids content in the extracts was expressed as g hyperoside equivalent (HE) and varied from 0.20 to 1.22 g/100 g dw. The highest level of tannins was found in *H. perforatum* (8.67 ± 0.02 g PE/100 g dw), followed by *H. maculatum* 7.06 ± 0.01 g PE/100 g dw). *H. elegans* has the lowest content of tannins (1.30 ± 0.01 g PE/100 g dw) and significantly low level of flavonoids (0.43 ± 0.03 g HE/100 g dw). *H. cerastoides* demonstrated the highest amount of flavonoids (1.22 ± 0.02 g HE/100 g dw) and moderate quantity of tannins (5.75 ± 0.01 g PE/100 g dw). Other species with high content of flavonoids were *H. tetrapterum* (1.13 ± 0.02 g HE/100 g dw) and *H. perforatum* (1.12 ± 0.01 g QE/100 g dw). The least quantity of flavanoids was found in *H. olympicum* (0.20 ± 0.03 g QE/100 g dw).

**Table 2:** Contents (g/100 g dw) of tannins and flavonoids in studied *Hypericum* species

Sample	Tannins	Flavonoids
*H. aucheri*	3.15 ± 0.01	0.74 ± 0.01
*H. barbatum*	2.89 ± 0.02	0.62 ± 0.02
*H. cerastoides*	5.75 ± 0.01	1.22 ± 0.02
*H. elegans*	1.30 ± 0.01	0.43 ± 0.03
*H. linarioides*	4.57 ± 0.01	0.94 ± 0.02
*H. maculatum*	7.06 ± 0.01	0.93 ± 0.02
*H. montbretii*	3.73 ± 0.09	1.04 ± 0.02
*H. olympicum*	3.28 ± 0.03	0.20 ± 0.03
*H. perforatum*	8.67 ± 0.02	1.12 ± 0.01
*H. richeri*	2.26 ± 0.01	0.69 ± 0.01
*H. rumeliacum*	4.53 ± 0.03	0.86 ± 0.01
*H. tetrapterum*	4.78 ± 0.02	1.13 ± 0.02
*H. umbellatum*	4.03 ± 0.02	0.76 ± 0.03

Results are represented as means ± standard deviation, *n* = 3

### DPPH, ABTS radical-scavenging and total antioxidant activity

The radical scavenging activity of *Hypericum* extracts (0.01 mg dw/ml) was compared with those of BHT and ascorbic acid at the same concentration and expressed as % of inhibition against DPPH and ABTS, respectively [Table 3]. *H. cerastoides* significantly quenched DPPH and ABTS (84.2% ± 0.3 and 90.2% ± 0.1), although it demonstrated a low total antioxidant activity (19.5 ± 0.8 μM TE/ g dw). These results well correlate with the high concentration of flavonoids and moderate level of tannins in the species. The scavenging ability of *H. perforatum* has significant values (77.6% ± 0.5 for DPPH and 81.2% ± 0.4 for ABTS) and corresponds to the presence of high quantity of phenolic compounds. *H. maculatum* and *H. olympicum* demonstrated significantly strong total antioxidant activity (101.8 ± 1 and 89.9 ± 0.2 μM TE/ g dw, respectively) compared to ascorbic acid and BHT. Although, *H. olympicum* has low content of tannins and flavonoids, the species showed high total antioxidant activity probably due to the presence of other compounds. This fact unambiguous proves that antioxidant potential is affected by many factors.

**Table 3:** DPPH, ABTS-radical scavenging and FRAP-activities of studied *Hypericum* species

Sample	DPPH %	ABTS %	FRAP μM TE/g dw
*H. aucheri*	58.2 ± 0.1	65.6 ± 0.2	46.5 ± 0.5
*H. barbatum*	31.9 ± 0.1	34.5 ± 0.2	9.45 ± 0.5
*H. cerastoides*	84.2 ± 0.3	90.2 ± 0.1	19.5 ± 0.8
*H. elegans*	25.9 ± 0.1	31.9 ± 0.2	9.8 ± 0.1
*H. linarioides*	54.2 ± 0.1	57.8 ± 0.4	29.6 ± 0.4
*H. maculatum*	56.2 ± 0.2	61.9 ± 0.2	101.8 ± 1
*H. montbretii*	71.2 ± 0.3	74.5 ± 0.3	59.8 ± 0.3
*H. olympicum*	58.8 ± 0.1	57.9 ± 0.1	89.9 ± 0.2
*H. perforatum*	77.6 ± 0.5	81.2 ± 0.4	32.4 ± 0.5
*H. richeri*	49.9 ± 0.1	55.7 ± 0.2	10.6 ± 0.4
*H. rumeliacum*	58.9 ± 0.2	65.2 ± 0.1	72.5 ± 0.5
*H. tetrapterum*	61.8 ± 0.2	68.3 ± 0.2	48.5 ± 0.5
*H. umbellatum*	49.1 ± 0.2	55.9 ± 0.2	61.35 ± 0.3
BHT	31.4 ± 0.2	77.3 ± 0.2	57.9 ± 0.5
Ascorbic acid	91.0 ± 0.6	96.2 ± 0.4	78.3 ± 0.7

Results are represented as means ± standard deviation, *n* = 3

### Antioxidant activity in linoleic acid system

**Figure 1:**
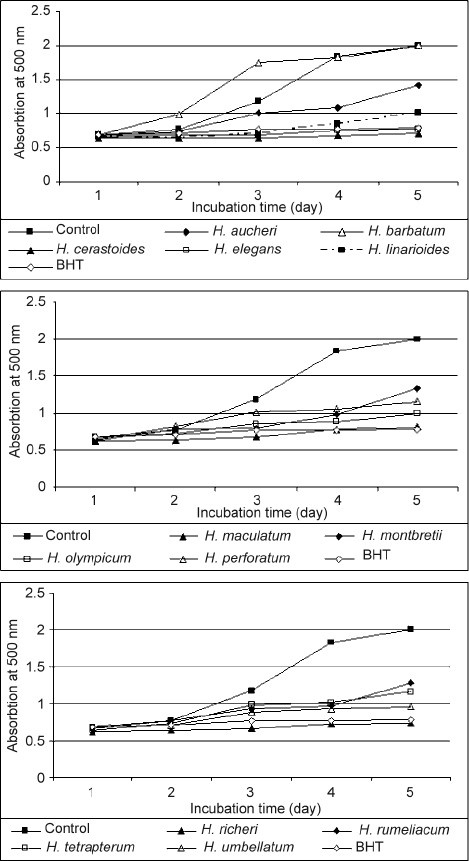
Antioxidant activity in linoleic acid system of studied *Hypericum* species

In the present study, the antioxidant activity of *Hypericum* extracts was determined by peroxidation of linoleic acid using the ferric thiocyanate method (FTC) [Figure 1]. During linoleic acid peroxidation, peroxides were formed and these compounds oxidized Fe^2+^ to Fe^3+^. The Fe^3+^ ion formed a complex with SCN^−^, which had a maximum absorbance at 500 nm.[19] Thus, a high absorbance value was an indication of high peroxide formation during the emulsion incubation. As shown in Figure 1, the absorbance of the control at 500 nm increased to a maximal value of 2 after 96 h, while *H. barbatum* was the only *Hypericum* sample, the absorption of which increased to 2. The highest significant diminution was demonstrated by *H. cerastoides* (0.7) followed by *H. richeri* (0.75) and *H. elegans* (0.78). However, the antioxidant activity of these *Hypericum* species was slightly less effective than that of BHT, a widely used commercial antioxidant. These results indicate that polar extracts from *Hypericum* species can significantly inhibit the peroxidation of linoleic acid and reduce the formation of hydroperoxide, thus implying that these plants are powerful natural antioxidants.

## CONCLUSION

The obtained results revealed that all tested *Hypericum* species exhibited radical scavenging. Furthermore, antioxidant activities of methanolic extracts from *H. cerastoides, H. perforatum* and *H. maculatum* were found to be the most potent. These species could be evaluated as rich sources of antioxidants.
